# Voxel-based meta-analysis of grey matter changes in Alzheimer’s disease

**DOI:** 10.1186/s40035-015-0027-z

**Published:** 2015-03-27

**Authors:** Wen-Ying Wang, Jin-Tai Yu, Yong Liu, Rui-Hua Yin, Hui-Fu Wang, Jun Wang, Lin Tan, Joaquim Radua, Lan Tan

**Affiliations:** Department of Neurology, Qingdao Municipal Hospital, School of Medicine, Qingdao University, No.5 Donghai Middle Road, Qingdao, Shandong Province 266071 China; College of Medicine and Pharmaceutics, Ocean University of China, Qingdao, 266011 China; Department of Neurology, Qingdao Municipal Hospital, Nanjing Medical University, Nanjing, 266071 China; Brainnetome Center, Institute of Automation, Chinese Academy of Sciences, Beijing, 100190 China; National Laboratory of Pattern Recognition, Institute of Automation, Chinese Academy of Sciences, Beijing, 100190 China; Department of Psychosis Studies, Institute of Psychiatry, King’s College London, London, UK; Research Unit, FIDMAG Germanes Hospitala’ries—CIBERSAM, Sant Boi de Llobregat, Barcelona, Spain

**Keywords:** Voxel-based morphometry (VBM), Alzheimer’s disease (AD), Grey matter (GM), Meta-analysis, Magnetic resonance imaging (MRI), Effect size signed differential mapping (ES-SDM)

## Abstract

**Background:**

Voxel-based morphometry (VBM) using structural brain MRI has been widely used for the assessment of impairment in Alzheimer’s disease (AD), but previous studies in VBM studies on AD remain inconsistent.

**Objective:**

We conducted meta-analyses to integrate the reported studies to determine the consistent grey matter alterations in AD based on VBM method.

**Methods:**

The PubMed, ISI Web of Science, EMBASE and Medline database were searched for articles between 1995 and June 2014. Manual searches were also conducted, and authors of studies were contacted for additional data. Coordinates were extracted from clusters with significant grey matter difference between AD patients and healthy controls (HC). Meta-analysis was performed using a new improved voxel-based meta-analytic method, Effect Size Signed Differential Mapping (ES-SDM).

**Results:**

Thirty data-sets comprising 960 subjects with AD and 1195 HC met inclusion criteria. Grey matter volume (GMV) reduction at 334 coordinates in AD and no GMV increase were found in the current meta-analysis. Significant reductions in GMV were robustly localized in the limbic regions (left parahippocampl gyrus and left posterior cingulate gyrus). In addition, there were GM decreases in right fusiform gyrus and right superior frontal gyrus. The findings remain largely unchanged in the jackknife sensitivity analyses.

**Conclusions:**

Our meta-analysis clearly identified GMV atrophy in AD. These findings confirm that the most prominent and replicable structural abnormalities in AD are in the limbic regions and contributes to the understanding of pathophysiology underlying AD.

## Introduction

Morphometric MRI studies have investigated focal structural abnormalities in brain tissue types, such as grey matter (GM) and white matter (WM), between groups of individuals using voxel-based morphometry (VBM). Briefly, the VBM approach presents lots of advantages such as fully automated, hypothesis-free, time-efficient, operator-independent and capable of investigating grey matter abnormalities across the whole brain than region of interest (ROI) analysis [1]. However, the major limitation of ROI-based techniques of morphometric brain changes is that this method requires a priori decision concerning which structures need to be evaluated [2]. Due to the small and heterogeneous samples of participants as well as substantial methodological differences between studies, results from VBM studies remain inconsistent and controversial [3]. As an intrinsical whole-brain technique, the VBM method exhibits comparable accuracy to manual volumetry and overcomes the limitations of ROI approach; therefore identifying consistent results from VBM studies of grey matter volume (GMV) in Alzheimer’s disease (AD) patients through meta-analysis is of particular significance.

AD is the most common type of dementia, the progressive neurodegenerative disorder, characterized by extensive neuronal and synaptic losses, as well as the presence of extracellular amyloid plaques, intracellular neurofibrillary tangles (NFTs) and brain volume reduction [4,5]. However, VBM studies of GMV in AD yield variable and conflicting evidence supporting these models; for example, some studies find regional grey matter atrophy mainly restricted to the medial temporal structures including bilateral hippocampus, amygdala and entorhinal cortex, as well as the posterior cingulate gyrus and medial thalamus [6,7], whereas a study found GM loss only in temporoparietal cortex [8].

Signed Differential Mapping (SDM) is a recently-developed statistical technique, which adopts and combines various positive features of activation likelihood estimate (ALE) and multilevel kernel density analysis (MKDA), in order to quantify the reproducibility of neuroimaging findings and generate insights difficult to observe in isolated studies [9]. Therefore, in the present study, we conducted a voxel-wisely meta-analysis VBM studies on AD using an SDM software to identify the consistent regional grey matter abnormalities in AD.

## Methods

### Search strategy

Systematic and comprehensive searches were conducted in PubMed (http://www.ncbi.nlm.nih.gov/pubmed/), ISI Web of Science (www.isiknowledge.com), Embase (www.embase.com/), and Medline databases (http://www.medline.com/) from 1995 to 25 June 2014 using the keywords “Alzheimer’s disease” OR “AD”, AND “voxel*”, “morphometry”,OR “vbm”. A hand searching was also performed in the reference lists of inclusion articles. The studies were considered for inclusion if they (1) reported VBM (GM density or volume) comparison between patients with AD and HC subjects; (2) reported whole brain results of changes in a stereotactic space in three dimensional coordinates (x, y, z); (3) used significance thresholds either corrected for voxel based multiple comparisons or uncorrected with spatial extent thresholds; and (4) were published in English with peer review. In studies that met the aforementioned inclusion criteria, the largest group size was selected if the data overlapped with the inter-subgroups or with another study. The studies were excluded if they suffered from at least one of the following deficiencies: (1) sufficient data could not be obtained even when more information was asked from the corresponding authors by phone or email; (2) there were fewer than nine subjects in either AD group or HC group; (3) the data overlapped with those of another publication; (4) there were uncorrected results and the spatial extent threshold was not reported; and (5) there was no HC group; (6) studies limit their analyses to specific ROI; (7) the patient-group included subgroups of “vascular” AD. and (8) it was not clear if the coordinates were in the Talairach or MNI (Montreal Neurological Institute) space (necessary for the text files in SDM software). The method used in the current study was in accordance with the Meta-analysis Of Observational Studies in Epidemiology (MOOSE) guidelines for meta-analyses of observational studies [10].

### Data extraction

The coordinates in each study were independently extracted by two neurologists (Wang WY and Yin RH) according to the ES-SDM method [9].

### Voxel-based meta-analysis (VBM)

The ROI is one of the most commonly used methods to address morphometric changes in the brain [2]. This method manually drew and calculated the brain regions of interest by investigators, then compared their volume in AD to HC. However, major limitations of the ROI approaches are that it requires a priori decision concerning which structures need to be evaluated and that regions showing abnormal GM volume might be part of a large ROI, or spread over different ROIs, thereby potentially reducing statistical power of the underlying morphological analysis [4]. This analysis was performed in a standard process using the SDM software (http://www.sdmproject.com/-software/) to compare the GM changes between the AD and HC groups. A systematic whole-brain voxel-based jackknife sensitivity analysis was performed to test the replicability of the results. All these processes were referred to the SDM Tutorial (http://sdmproject.com/software/Tutorial.pdf) and publications. The full-width at half maximum (FWHM) in SDM are set at 20 mm because in previous simulations it has been found to have an excellent control of false positives results in a preprocessing step [9]. The statistical threshold was set to be a p-value of < 0.005 without correction for false discovery rate (FDR) as this was found to optimally balance sensitivity and specificity [9]. Residual heterogeneity was not significant (τ = 0.154, Q = 83.926, df = 28, P = 1.79 × 10^−7^). The SDM software editor was also contacted by email when necessary.

## Result

### Included studies and sample characteristics

**Figure 1:**
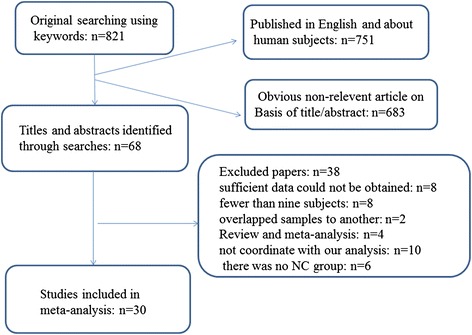
**Flowchart describing the approach used to identify all eligible studies of meta-analysis.**

The initial literature search identified 821 potentially relevant articles, of which 68 met the inclusion criteria. After full text screening, 38 articles were excluded for different reasons (Figure 1). Finally, 30 [7,11-39] articles published between 1995 and 2014 met the selection criteria and had accessible information concerning grey matter changes between AD and HC. The clinical and demographic data of participants in all included studies are presented in Table 1. The technical details of the included studies are shown in Table 2. A total of 960 people with AD and 1195 HC were included. In each study, no statistically significant difference was found in age, gender between the AD and HC, as the original studies were already well matched in this respect. Sensitivity analysis was first used and no outliers were found in this study.

**Table 1:** **Demographic and clinical characteristics of VBM studies for GMV on AD in meta-analysis**

**Study**	**Number**		**Age**		**Education**		**MMSE**		**Diagnostic criteria**
	**(Female)**		**(Years)**		**(Years)**				
	**AD**	**NC**	**AD**	**NC**	**AD**	**NC**	**AD**	**NC**	
Guo2014 [11]	35 (23)	27 (11)	72.4 (8.5)	69.2 (6.5)	NA	NA	19.7 (4.1)	28.9 (1.0)	ICD-10 criteria
Teipel2012 [30]	137 (79)	143 (72)	72.5 (8.3)	69.2 (5.9)	10.2 (3.3)	13.1 (3.8)	20.6 (5.3)	28.8 (1.1)	NINCDS-ADRDA
Rami2012 [12,62]	32	24	75.5 (5.5)	71.4 (6.6)	8.3 (2.9)	9.0 (4.7)	22.5 (3.3)	28.1 (1.4)	NINCDS-ADRDA
Gili2011 [29]	11 (4)	10 (3)	71.9 (7.9)	64.1 (10.5)	9.9 (4.9)	14.3 (3.4)	19.7 (4.5)	28.34 (2.0)	NINCDS-ADRDA
Lehmann2011 [13]	30 (16)	50 (33)	69.2 (8.9)	63.7 (9.6)	NA	NA	NA	NA	NINCDS-ADRDA
Serra2010 [14]	9 (6)	13 (4)	72.4 (7.5)	64.1 (10.5)	9.9 (4.9)	14.3 (3.4)	18.2 (4.4)	28.9 (1.3)	NINCDS-ADRDA
Guo2010 [23]	13 (7)	14 (8)	72.1 (6.5)	70.4 (3.5)	NA	NA	18.5 (3.5)	28.5 (0.6)	NINCDS-ADRDA
Ibrahim2009 [22]	20 (11)	23 (18)	73.67 (7.58)	66.70 (5.82)	13.10 (2.95)	14.22 (2.36)	15.60 (7.20)	29.13 (0.99)	NINCDS-ADRDA
Kanda2008 [15]	20	20	65.0	65.2	NA	NA	17.5	29.0	NINCDS-ADRDA
Whitwell2007 [5,16]	38 (22)	38 (22)	65.3 (6.9)	65.9 (7.0)	12.5	14.0	17.0	29.0	NINCDS-ADRDA
Hamalainen2007 [24]	15 (10)	21 (17)	73.1 (6.7)	71.2 (4.9)	8.2 (2.7)	7.9 (2.9)	21.7 (3.7)	27.7 (2.0)	NINCDS-ADRDA
DiPaola2007 [18]	18 (14)	18 (14)	64.3 (10.2)	65.4 (10.6)	8.8 (4.2)	8.1 (2.8)	16.9 (4.3)	29.0 (1.6)	NINCDS-ADRDA
Samuraki2007 [28]	39 (20)	73 (36)	68.03 (8.76)	66.78 (8.34)	NA	NA	22.3 (3.3)	29.2 (0.8)	NINCDS-ADRDA
Rabinovici2007 [17]	11 (6)	40 (23)	64.5 (9.7)	63.5 (5.8)	16.5 (2.9)	17.4 (2.4)	19.9 (6.9)	29.7 (0.5)	NINCDS-ADRDA
Bozzali2006 [26]	22 (11)	20 (13)	67.9 (7.6)	65.8 (6.8)	NA	NA	19.8 (4.1)	27.3 (1.2)	NINCDS-ADRDA
Shiino2006 [27]	40 (21)	88 (48)	71.1 (9.7)	68.7 (8.7)	NA	NA	18.03 (3.91)	29.09 (1.47)	NINCDS-ADRDA
Ishii2005 [19]	30 (22)	30 (22)	66.8 (7.0)	66.8 (7.9)	NA	NA	14.7 (5.4)	24.0 (2.2)	NINCDS-ADRDA
Hirata2005 [7]	61 (29)	82 (43)	70.6 (8.4)	70.1 (7.7)	NA	NA	26.0 (1.5)	28.7 (1.5)	NINCDS-ADRDA
Boxer2003 [20]	11 (8)	15 (7)	69.6 (8.2)	65.1 (8.3)	16.3 (3.8)	16.6 (3.9)	20.2 (7.3)	29.5 (0.5)	NINCDS-ADRDA
Frisoni2002 [21]	29 (23)	26 (17)	74 (9)	69 (8)	7 (4)	8 (3)	21 (4)	29 (1)	NINCDS-ADRDA
Ohnishi2001 [25]	26 (15)	47 (16)	72.1 (1.1)	28.5 (6.0)	NA	NA	20.7 (3.1)	NA	NINCDS-ADRDA
Matsuda2002 [34]	15 (4)	25 (9)	71.1 (7.1)	71.2 (7.3)	NA	NA	21.5 (2.9)	29.5 (0.6)	DSM-IV
Busatto2003 [35]	14 (5)	14 (6)	72.2 (7.2)	69.4 (5.9)	9.1 (4.2)	7.1 (4.7)	20.7 (3.1)	29.1 (0.5)	NINCDS-ADRDA
Raji2009 [31]	33 (20)	169 (96)	82.8 (5.16)	77.57 (3.62)	NA	NA	NA	NA	NINCDS-ADRDA
Honea2009 [32]	61 (37)	56 (33)	74.3 (6.3)	73.3 (6.2)	15.3 (3.3)	16.4 (2.2)	26.2 (3.7)	29.4 (0.8)	NINCDS-ADRDA
Brenneis2004 [36]	10 (3)	10 (6)	73.1 (7.6)	65.1 (8.1)	NA	NA	17.4 (7.9)	28.8 (1.6)	DSM-IV
Hirao2006 [39]	61 (29)	61 (31)	70.6 (8.4)	70.2 (7.3)	NA	NA	26.0 (1.5)	28.7 (1.5)	NINCDS-ADRDA
Brambati2009 [33]	10 (5)	13 (8)	71.5 (5.9)	75.0 (5.0)	12.6 (4.7)	14.9 (5.0)	22.5 (2.3)	29.1 (1.2)	NINCDS-ADRDA
Baxter2006 [38]	15 (4)	15 (8)	75.5 (7.8)	76.4 (7.9)	14.7 (2.9)	15.3 (2.9)	14–28	28.5 (1.1)	NINCDS-ADRDA
Zahn2005 [37]	10 (6)	10 (5)	66.5 (8.9)	65.8 (7.8)	NA	NA	23.6 (2.8)	NA	NINCDS-ADRDA

Key: AD, Alzheimer’s disease; NC, normal cognition subjects; MMSE, the Mini Mental State Examination; NA, not available; VBM, voxel-based morphometry; GMV, Grey matter volume; NINCDS-ADRDA, National Institute of Neurological and Communicative Disorders and Stroke/Alzheimer’s Disease and Related Disorders Association; DSM, Diagnostic and Statistical Manual of Mental Disorders; ICD-10 criteria, International Classification of Diseases, 10th Revision.

**Table 2:** **Technique details of VBM studies for GMV on AD in meta-analysis**

**Study**	**Scanner (T)**	**Software**	**FHWH (mm)**	**P-value**	**Coordinates**
Guo2014 [11]	3	SPM8	6	P < 0.05 (FDR-corrected)	14
Teipel2012 [30]	1.5 (3)	SPM8	8	P < 0.001, uncorrected for multiple comparisons	22
Rami2012 [12,62]	3	SPM5	8	P < 0.0001 (uncorrected)	4
Gili2011 [29]	3	SPM5	12	P < 0.001 uncorrected	9
Lehmann2011 [13]	1.5	SPM5	6	Multiple-comparison correction FDR < 0.05	1
Serra2010 [14]	3	SPM5	12	P < 0.05 (FEW corrected)	17
Guo2010 [23]	3	SPM5	8	P < 0.05 (FDR Corrected)	16
Ibrahim2009 [22]	1.5	SPM5	10	p-value < 0.005 with the FDR corrected	15
Kanda2008 [15]	1.5	SPM2	12	P < 0.01, corrected	7
Whitwell2007 [5,16]	1.5	SPM5	8	P < 0.05, corrected for multiple comparisons	1
Hamalainen2007 [24]	1.5	SPM5	12	P < 0.05, corrected	19
DiPaola2007 [18]	1.5	SPM5	10	P < 0.05 (FWE corrected)	18
Samuraki2007 [28]	1.5	SPM2	12	P < 0.001,correct for multiple comparisons	5
Rabinovici2007 [17]	1.5	SPM2	12	P < 0.05 (FWE- corrected)	19
Bozzali2006 [26]	1.5	SPM2	12	P < 0.05 corrected	19
Shiino2006 [27]	1.5	SPM99	12	P < 0.05 corrected	16
Ishii2005 [19]	1.5	SPM5	12	P < 0.05, corrected	3
Hirata2005 [7]	1	SPM5	12	P < 0.001, correction for multiple non-independent comparisons	2
Boxer2003 [20]	1.5	SPM5	12	P < 0.05,corrected for multiple comparisons	3
Frisoni2002 [21]	1.5	SPM99	8	P < 0.05, corrected for multiple comparisons	34
Ohnishi2001 [25]	1.0	SPM96	12	P < 0.001, correct for multiple comparisons	2
Matsuda2002 [34]	1.0	SPM99	12	P < 0.05 correct for multiple comparisons	13
Busatto2003 [35]	1.5	SPM99	8	P < 0.001, uncorrected	9
Raji2009 [31]	1.5	SPM2	10	P < 0.05 (FDR Corrected)	5
Honea2009 [32]	3.0	SPM5	10	P < 0.05, FWE corrected	13
Brenneis2004 [36]	1.5	SPM99	8 and 10	P < 0.05 corrected for small volumes	14
Hirao2006 [39]	1.0	SPM2	12	P < 0.001; corrected for multiple comparisons	2
Brambati2009 [33]	3.0	SPM5	8	p < 0.001 uncorrected	17
Baxter2006 [38]	1.5	SPM2	12 and 8	p-values <0.0001 uncorrected	6
Zahn2005 [37]	1.5	SPM2	8	P < 0.001 uncorrected	4

Key: AD, Alzheimer’s disease; FDR, false discovery rate; FEW, family-wise error; FWHM, full width at half-maximum; SPM, Statistical Parametric Mapping; T, Tesla; VBM, voxel-based morphometry; GMV, grey matter volume.

### Global GM volumes

This analysis was not carried out because of the small number of studies with a detailed global GM density or volume.

### Regional differences

**Figure 2:**
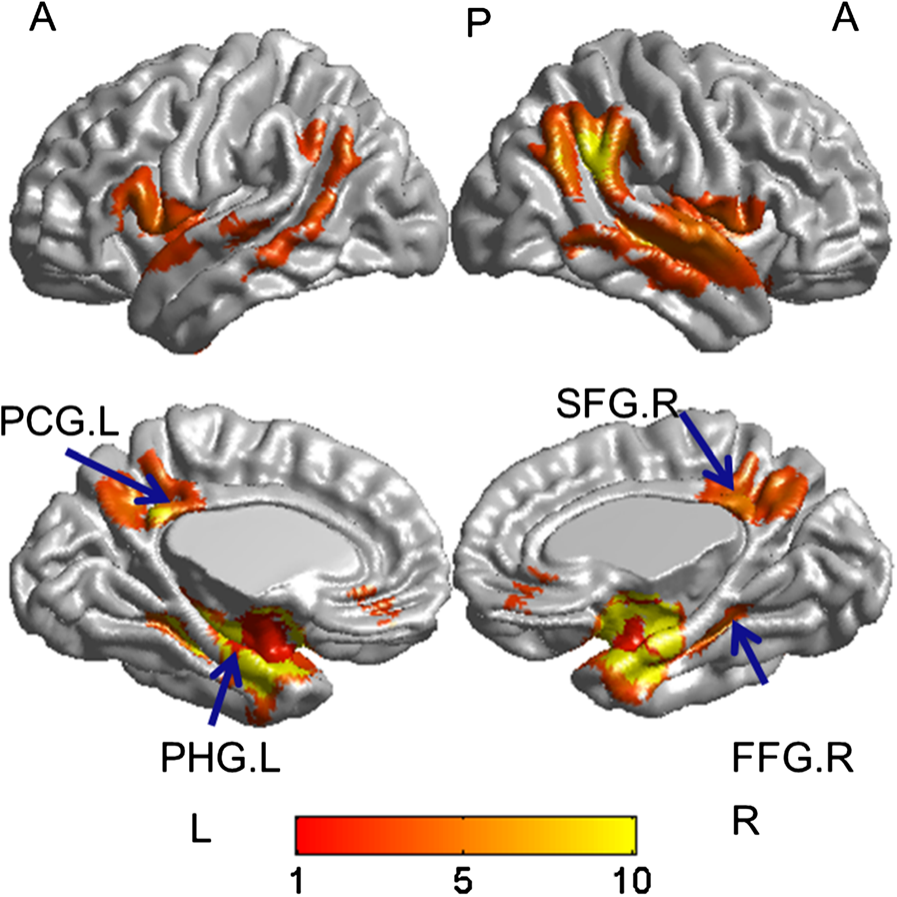
**Brat v1.0 (** **www.brainnetome.org/brat** **) software was used to visualize the anatomical distribution of grey matter atrophy in AD.** PHG.L: left parahippocampl gyrus; PCG.L: left posterior cingulate gyrus; SFG.R: right superior frontal gyrus; FFG.R: right fusiform gyrus; L: left, R: right., A: anterior, P: posterior.

The included studies reported GM reductions at 334 coordinates in AD compared with NC. A group comparison of AD patients and HC was carried out. AD patients had considerable smaller GMV in the limbic regions (left parahippocampl gyrus and left posterior cingulate gyrus). In addition, there were GM decreases in right fusiform gyrus and right superior frontal gyrus (as shown in Table 3 and Figure 2) in patients with AD from the SDM map threshold of P < 0.005 with voxels > 10. The patients with AD had no significant GM increase in any region compared with the HC subjects in all included studies.

**Table 3:** **Regional differences in grey matter volume between individuals with AD and HC**

**Region**	**Maximum**				**Cluster**	**Jackknife sensitivity analysis (combination of studies detecting the differences)**
	**MNI coordinates x y z**	**SDM value**	**P value**	**Number of voxels**	**Clusters breakdown (no. of voxels)**	
Left parahippocampl gyrus, BA36	−30,-10,-28	−6.544	~0	3440	Left parahippocampal gyrus (676)	30 out of 30
					Left insula (515)	
					Left temporal gyrus (879)	
					Left hippocampus (706)	
					Left inferior frontal gyrus (450)	
					Left rolandic operculum (214)	
Right fusiform gyrus	34,-8,-30	−6.257	~0	5838	Right temporal gyrus (3300)	29 out of 30
					Right parahippocampal gyrus (813)	
					Right hippocampus (521)	
					Right rolandic operculum (447)	
					Right insula (757)	
Left posterior cingulate gyrus, BA 23	−8,-48,32	−3.880	~0	1866	Left /Right precuneus (1056)	30 out of 30
					Left/Right posterior cingulate gyrus (358)	
					Left/Right median cingulate/paracingulate gyri (452)	
Right superior frontal gyrus, medial orbital, BA 11	4,34,-12	−3.362	0.000474795	221	Left/Right superior frontal gyrus, medial orbital (84)	28 out of 30
					Left /Right gyrus rectus (23)	
					Left anterior cingulate/paracingulate gyri (18)	

Regions identified by meta-analysis of coordinates from Twenty one studies (voxelwise p < 0.005 and FWHM 20 mm).

Key: AD, Alzheimer’s disease; HC, healthy controls; MNI, Montreal Neurological Institute; SDM, signed differential mapping; GMV, Grey matter volume; BA, Brodmann area.

### Sensitivity analysis

A whole-brain voxel-based jackknife sensitivity analysis was conducted to test the replicability of the results. This consists of repeating the main statistical analysis 30 times but systematically removing one different study each time to ensure that no single study will bias the combined results and recalculating the stability of the remaining studies. As shown in Table 3, the whole brain jackknife sensitivity analysis indicated a GM reduction in left parahippocampal gyrus and left posterior cingulate gyrus highly replicable because they were preserved throughout all of the 30 combinations of studies. Grey matter decreases in right fusiform gyrus failed to emerge in one of the study and right superior frontal gyrus failed to emerge in two of the study.

### Analyses of subgroups

The above results were highly reproducible when the analyses were repeated and limited to 12-mm smoothing kernel or 1.5 T MRI scanner to remove the potential confounding effects of methodological differences.

### Meta-regression

The meta-regression analysis showed that the higher neuropsychological test scores in AD (Mini Mental State Examination (MMSE) scores- used as a concise screening tool that assesses the severity of cognitive impairment and reflects cognitive in the progression of AD [40], available in all the studies) was associated with decreased grey matter volumes in the left parahippocampal gyrus ( −18,-16, −28; SDM-Z = −3.963; voxels = 490; P = 3.0965 × 10^−5^).

## Discussion

To our knowledge, this is the first meta-analysis of voxel-based morphometry studies of grey matter volume in AD and HC subjects using ES-SDM software. The present voxel-wise meta-analysis mainly found that patients with AD have regional GM volume reductions in the limbic regions (left parahippocampl gyrus and left posterior cingulate gyrus). In addition, there were GM decreases in right fusiform gyrus and right superior frontal gyrus, medial orbital. The results remained largely unchanged when jackknife sensitivity analysis was performed. This indicates that the results were robust and highly replicable.

It has been widely accepted that at rest state, important brain areas-posterior cingulate cortex combines precuneus, lateral temporal cortex, medial prefrontal cortex, and inferior parietal lobule organized into a functionally relevant networks, the “default mode network” (DMN) [41], which is correlated with episodic memory functioning. Several studies have demonstrated that AD is associated with DMN resting state functional MRI disruptions compared to HC, which is marked by abnormalities in structural interactions and functional connectivity [42-44]. In a previous study using single photon emission computed tomography (SPECT), Pagani et al. proposed that posterior cingulate cortex covaried with the left lateral parietal lobe [45]. In addition, Jacobs et al. demonstrated that grey matter atrophy in inferior parietal lobule was connected to the prefrontal cortices [46]. Recently, Wang and colleagues employing Bayesian network models and integrating grey matter volume information from multiple brain regions found increased correlations from Left inferior temporal cortex to Left hippocampus, Left hippocampus to Right inferior temporal cortex, Right hippocampus to Right inferior temporal cortex, and Right inferior parietal cortex to Posterior cingulate cortex in AD patients [47]. The morphological changes in the grey matter in different brain regions abide by covariance pattern, reflecting the DMN network attributes of the human brain, and also suggest that the atrophy of these structures is not independent, but that primary neurodegeneration in one of the structures that could lead to secondary degeneration of regions connected to it.

A previous reported meta-analysis of ALE structural MRI studies found evidence of volume reductions in the medial temporal lobes (MTL) (including entorhinal cortex, hippocampus, parahippocampus, amygdala and uncus), temporal, frontal and cingulate cortices [48]. This is in line with our meta-analysis. However , our study did not found volume reductions in parietal and insular cortices. This may be due to the inclusion of recently available data and the improvement of meta-analytic method.

Grey matter differed in AD and Dementia with Lewy bodies (DLB) when compared the result of the meta-analysis using the SDM methodologies. AD was characterized by GMV decreased in the MTL but not the lateral temporal lobe are coincident with the recently published meta-analysis showed that medial temporal lobe structures were relative preserved in DLB compared with AD [49]. In AD, the MTL have been proved associated with the degree of memory, regions which are involved in encoding and retrieval of episodic and spatial memory [50,51]. Pathological investigations in AD confirmed our findings, where the neurofibrillary tangles and amyloid plaques observed from the beginning [52], and subsequently affects the posterior limbic system due to its close connections to the posterior sector of the cingulate gyrus. So far we could not find the Tau pathology are connected with GMV loss in AD in MTL. Furthermore, the postmortem autopsy also have been testified it as cardinal structures affected with loss of neurons in patients with AD [53] of which may explanation of the MTL atrophy and supports our results. A previous meta-analysis of structural and functional imaging studies also revealed consistent volumetric reduction within the MTL was the most sensitive measure to identify AD in patients with a duration of illness greater than 4 years [54]. The volumetric reductions seen in this region may help explain reports of episodic memory loss in early stages of AD.

One of the key findings of the present study is the GM volume reduction in the left parahippocampal gyrus, the most important cortical input and output region of the hippocampus and mediates corticohippocampal communication. An important study by Burgmans et al. found the association with memory decline is larger in the posterior parahippocampus than in the hippocampus and entorhinal cortex [55], and parahippocampal gyrus atrophy in AD are related to patients’ anterograde memory impairments. In addition, the finding of robust GM loss in the parahippocampal gyrus is in line with one previous ROI-based quantitative volumetric MRI study [56]. Previous histological studies have already revealed that entorhinal cortex as the earliest neuropathological changes in AD patients, which is the anterior part of the parahippocampal gyrus [57]. Abnormalities in this region is also confirmed by previous researches through other neuroimaging methods such as functional MRI (fMRI) [58], technetium (Tc-99 m) hexamethylpropyleneamine oxime (TC-99 m HMPAO) SPECT [59] and Pittsburgh Compound B [60].

Our meta-analysis revealed grey matter reductions in left posterior cingulate gyrus. Evidence from the positron emission tomography (PET) studies using [11C]PiB (Pittsburgh compound B) binds to amyloid, have identified that this region was related to the disease process [61]. The finding of robust GM loss in parietal regions is in line with a recent fMRI study, which have found atrophy in this region in the very early progression of AD [62]. This result is also in accordance with previous Fluorodeoxyglucose (FDG) PET studies which reported metabolic decline in the posterior cingulate cortex of patients with AD [63]. In addition, using dynamic susceptibility contrast magnetic resonance imaging, Hauser and colleagues have detected that the posterior cingulate gyrus perfusion was significantly decreased in patients with AD compared to patients with MCI or HC [64]. Likewise, Yoshida et al. revealed decreased regional cerebral blood flow and regional cerebral protein synthesis in this region. The probable explanation of the posterior cingulate gyrus atrophy is that neuronal atrophy and the fibrillary amyloid deposition which need further investigation.

The current study has a number of strengths. The most importantly utilized SDM methodologies, a well-validated, automated method of meta-analyzing data from multiple VBM studies using the reported peak coordinates to recreate (to a limited extend) the original maps, thus accounting for both positive and negative differences [9]. This technique has already been successfully applied in a number of previous meta-analysis of VBM studies on several neurologic and neuropsychological disorders such as amyotrophic lateral sclerosis [65], obsessive-compulsive disorder [66], bipolar disorder [67] and DLB [49]. The ES-SDM that we used in this study is a new version of the SDM meta-analytic method featuring two methodological improvements: combining peak coordinates and statistical parametric maps and use of well-established statistics accounting for within- and between-study variance [9]. The new version-ES-SDM has been proved to be valid and superior to previous coordinate-based meta-analytical methods such as ALE and the default settings, and also optimizes the sensitivity while protecting against the false positives [9].

### Limitations

There are several methodological limitations of this study, some of which are inherent to all meta-analytical approaches. One limitation is the accuracy of the results, because peak-based meta-analyses are based on pooling of stereotactic coordinates rather than on raw statistical brain maps, and this may lead to less accurate results. Nevertheless, obtaining and analysing the raw images from these studies is logistically and technically difficult. Second, methodological differences of VBM studies, for instance different preprocessing protocols (traditional or optimized), smoothing kernels, and statistical thresholding methods cannot be entirely ruled out even if a subgroup analysis was performed. Third, as mentioned above, our regression analyses should be taken cautiously because they included a small number of studies and variability in the data was limited. Fourth, several of the included studies reported grey matter density rather than volume. The mean density of the GM is derived from the percentage of absolute GM volume divided by total brain volume, which might result in different locations of deficit areas from results achieved by VBM measurements of GM volume. The meta-analysis will subsequently influence the results by different locations. Finally, although voxel-wise meta-analytical methods provide excellent control for false-positive results, it is difficulty to avoiding false-negative results completely. Since the SDM approach does not use effect sizes for non-significant changes (e.g., those that have p <0.005 and do not survive correction for multiple comparisons), in some regions with some trends towards significant or even just differences that appear not to be significant because of the effect size, it might cause false negative findings.

## Conclusion

The results of meta-analysis implicate regional grey matter reduction in AD is a functionally relevant networks (DMN) of prefrontal, limbic, temporal regions involved in the episodic memory functioning and attentional processing compared with HC. A better understanding of the neural network implicated in AD may inform the diagnosis and treatment of this condition in the future.
